# 2-Hy­droxy-*N*′-methyl­benzohydrazide

**DOI:** 10.1107/S160053681203468X

**Published:** 2012-08-11

**Authors:** Xinwen Zhang

**Affiliations:** a College of Chemistry and Material Science, South-Central University for Nationalities, Wuhan 430074, People’s Republic of China

## Abstract

In the title mol­ecule, C_8_H_10_N_2_O_2_, there is an intra­molecular hydrogen bond involving the hy­droxy group and the O atom of the carbonyl group. The dihedral angle between the benzene ring and the amide fragment is 87.16 (10)°. The C—N—N—C torsion angle is 88.87 (18)°. In the crystal, N—H⋯N and N—H⋯O hydrogen bonds connect mol­ecules into chains along [100]. In addition, there is a weak C—H⋯π inter­action.

## Related literature

For applications of related materials, see: Zhang *et al.* (2012 ▶); Jin *et al.* (2011 ▶). For the preparation of the title compound, see: Li *et al.* (2001 ▶). For a related structure, see: Jin (2007 ▶).
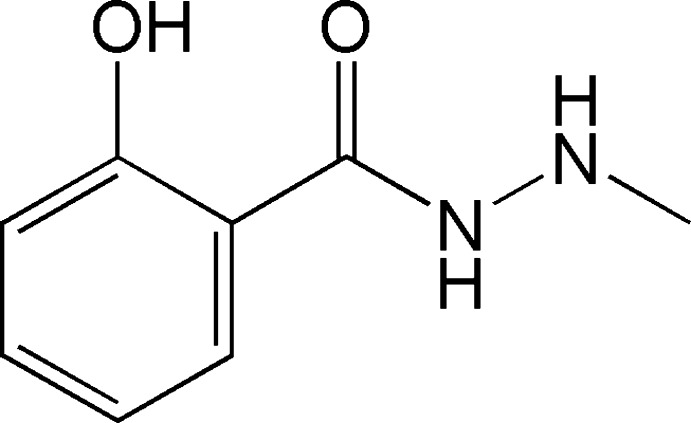


## Experimental

### 

#### Crystal data

C_8_H_10_N_2_O_2_
*M* *_r_* = 166.18
Monoclinic, 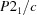

*a* = 7.4863 (10) Å
*b* = 14.706 (2) Å
*c* = 7.7232 (11) Å
β = 96.898 (2)°
*V* = 844.1 (2) Å^3^
*Z* = 4
Mo *K*α radiation
μ = 0.10 mm^−1^
*T* = 294 K
0.30 × 0.20 × 0.20 mm

#### Data collection

Bruker SMART CCD diffractometer
Absorption correction: multi-scan (*SADABS*; Sheldrick, 1996 ▶) *T*_min_ = 0.972, *T*_max_ = 0.981
6478 measured reflections
1837 independent reflections
1381 reflections with *I* > 2σ(*I*)
*R*_int_ = 0.045

#### Refinement

*R*[*F* ^2^ > 2σ(*F* ^2^)] = 0.048
*wR*(*F* ^2^) = 0.129
*S* = 1.03
1837 reflections
119 parameters
H atoms treated by a mixture of independent and constrained refinement
Δρ_max_ = 0.16 e Å^−3^
Δρ_min_ = −0.26 e Å^−3^

### 

Data collection: *SMART* (Bruker, 2001 ▶); cell refinement: *SAINT* (Bruker, 2001 ▶); data reduction: *SAINT*; program(s) used to solve structure: *SHELXS97* (Sheldrick, 2008 ▶); program(s) used to refine structure: *SHELXL97* (Sheldrick, 2008 ▶); molecular graphics: *SHELXTL* (Sheldrick, 2008 ▶); software used to prepare material for publication: *SHELXTL*.

## Supplementary Material

Crystal structure: contains datablock(s) global, I. DOI: 10.1107/S160053681203468X/lh5501sup1.cif

Structure factors: contains datablock(s) I. DOI: 10.1107/S160053681203468X/lh5501Isup2.hkl

Supplementary material file. DOI: 10.1107/S160053681203468X/lh5501Isup3.cml

Additional supplementary materials:  crystallographic information; 3D view; checkCIF report

## Figures and Tables

**Table 1:** Hydrogen-bond geometry (Å, °) *Cg* is the centroid of the C1–C6 ring.

*D*—H⋯*A*	*D*—H	H⋯*A*	*D*⋯*A*	*D*—H⋯*A*
O1—H1*B*⋯O2	0.91 (2)	1.72 (2)	2.5535 (15)	150 (2)
N1—H1*A*⋯N2^i^	0.859 (19)	2.16 (2)	2.9415 (18)	151.9 (15)
N1—H1*A*⋯N1^i^	0.859 (19)	2.619 (18)	3.1403 (18)	120.4 (13)
N2—H2*A*⋯O2^ii^	0.884 (18)	2.253 (17)	2.9866 (16)	140.3 (14)
C4—H4⋯*Cg* ^iii^	0.93	2.83	3.6713 (13)	152

Symmetry codes: (i) 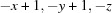; (ii) 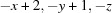; (iii) 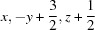.

**Fig. 1.**
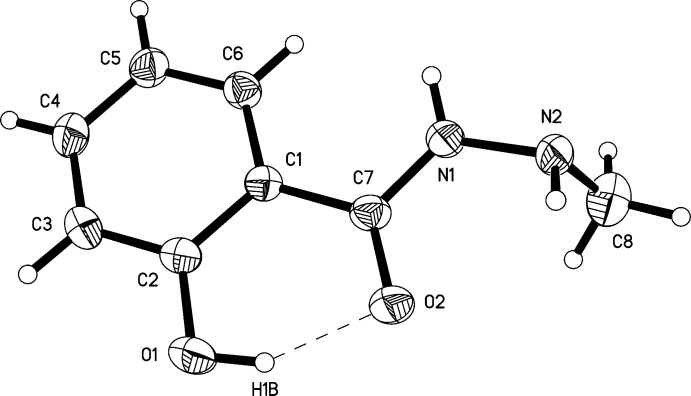
The molecular structure of (I), showing 30% probability displacement ellipsoids. The dashed lines indicates a hydrogen bond.

**Fig. 2.**
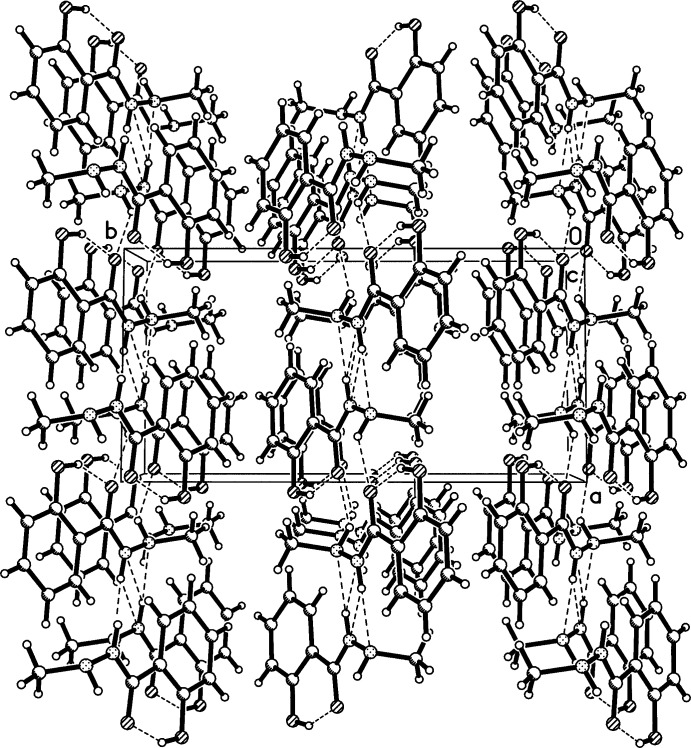
Packing diagram for (I). The hydrogen bonds are indicated by dashed lines.

## supplementary crystallographic information

### Comment

Materials such as the title compound and the recently determined crystal structure of 4-(5-bromo-2-hydroxybenzoyl)thiosemicarbazide (Jin, 2007) are potentially important ligands and intermediates (Zhang *et al.*, 2012; Jin *et al.*, 2011). Part of our studies is to find new methods to synthesize derivatives of saylicylic acid and study their structures and activities. In this paper, the X-ray crystal structure determination of the title compound (I) is reported.

The molecular structure of the title compound is shown in Fig. 1. The geometric parameters of (I) agree with those related in the structure published by Jin (2007). The atoms C1—C7/O1/O2 are essentially co-planar with an r.m.s deviation of 0.016Å. There is intramolecular hydrogen bond involving the hydroxy group and the O atom of the carbonyl group. In the crystal, N—H···N and N—H···O hydrogen bonds connect molecules into one-dimensional chains along [100] (see Table 1 and Fig. 2). In addition, there is a weak intermolecular C—H···π interaction. There are some intermolecular contacts which are shorter than the sums of the van der Waals radii of the atoms involved i.e. N1···N2^iv^ [2.9415 (18) Å], O2···N2^v^ [2.9866 (17) Å] and C6···C6^vi^ [3.346 (2) Å] [symmetry code (iv): 1 - *x*,-*y*,1 - *z*; (v): -*x*,-*y*,1 - *z*; (vi): 1 - *x*,-*y*,-*z*].

### Experimental

Methyl salicylate (15.2 g, 0.10 mol) and methylhydrazine in aqueous solution (23.0 g, 0.20 mol) were mixed at 273 K and stirred for 1 h. The reaction mixture was slowly warmed to 338 K and refluxed for a further 12 h. After the resulting mixture was concentrated under reduced pressure, the residue was adjusted to pH 8 with acetic acid. After staying for 1 h in a refrigerator, the resulting precipitate was filtered and rinsed with ethyl ether. A white solid formed was recrystallized to give 7.0 g (42% yield) of *N*-methyl-salicylhydrazide. A block-like crystal suitable for X-ray analysis was grown from a solution of the title compound in methanol at room temperature by slow evaporation.

### Refinement

The hydroxy H atom was located in a difference Fourier map and refined isotropically [O—H = 0.91 (2) Å] with U_iso_(H) = 1.5U_eq_(O). The H atoms bonded to N atoms were located in a difference Fourier map and refined isotropically (N—H = 0.859 (19) and 0.884 (18) Å) with U_iso_(H) = 1.2U_eq_(N). All other H atoms were included in a riding-model approximation, with C—H distances of 0.93 (aromatic H atoms) and 0.96 Å (methyl atoms). The isotropic displacement parameters were set to 1.2*U*_eq_(C) for the aromatic H atoms and to 1.5*U*_eq_(C) for the methyl H atoms.

### Crystal data

**Table:**

C_8_H_10_N_2_O_2_	*F*(000) = 352
*M**_r_* = 166.18	*D*_x_ = 1.308 Mg m^−^^3^
Monoclinic, *P*2_1_/*c*	Mo *K*α radiation, λ = 0.71073 Å
Hall symbol: -P 2ybc	Cell parameters from 1880 reflections
*a* = 7.4863 (10) Å	θ = 2.7–26.5°
*b* = 14.706 (2) Å	µ = 0.10 mm^−^^1^
*c* = 7.7232 (11) Å	*T* = 294 K
β = 96.898 (2)°	Block, colorless
*V* = 844.1 (2) Å^3^	0.30 × 0.20 × 0.20 mm
*Z* = 4	

### Data collection

**Table:**

Bruker SMART CCD diffractometer	1837 independent reflections
Radiation source: fine-focus sealed tube	1381 reflections with *I* > 2σ(*I*)
Graphite monochromator	*R*_int_ = 0.045
φ and ω scans	θ_max_ = 27.0°, θ_min_ = 2.7°
Absorption correction: multi-scan (*SADABS*; Sheldrick, 1996)	*h* = −7→9
*T*_min_ = 0.972, *T*_max_ = 0.981	*k* = −16→18
6478 measured reflections	*l* = −9→9

### Refinement

**Table:**

Refinement on *F*^2^	Primary atom site location: structure-invariant direct methods
Least-squares matrix: full	Secondary atom site location: difference Fourier map
*R*[*F*^2^ > 2σ(*F*^2^)] = 0.048	Hydrogen site location: inferred from neighbouring sites
*wR*(*F*^2^) = 0.129	H atoms treated by a mixture of independent and constrained refinement
*S* = 1.03	*w* = 1/[σ^2^(*F*_o_^2^) + (0.0724*P*)^2^] where *P* = (*F*_o_^2^ + 2*F*_c_^2^)/3
1837 reflections	(Δ/σ)_max_ = 0.001
119 parameters	Δρ_max_ = 0.16 e Å^−^^3^
0 restraints	Δρ_min_ = −0.26 e Å^−^^3^

### Special details

**Table:**

Geometry. All e.s.d.'s (except the e.s.d. in the dihedral angle between two l.s. planes) are estimated using the full covariance matrix. The cell e.s.d.'s are taken into account individually in the estimation of e.s.d.'s in distances, angles and torsion angles; correlations between e.s.d.'s in cell parameters are only used when they are defined by crystal symmetry. An approximate (isotropic) treatment of cell e.s.d.'s is used for estimating e.s.d.'s involving l.s. planes.
Refinement. Refinement of *F*^2^ against ALL reflections. The weighted *R*-factor *wR* and goodness of fit *S* are based on *F*^2^, conventional *R*-factors *R* are based on *F*, with *F* set to zero for negative *F*^2^. The threshold expression of *F*^2^ > σ(*F*^2^) is used only for calculating *R*-factors(gt) *etc*. and is not relevant to the choice of reflections for refinement. *R*-factors based on *F*^2^ are statistically about twice as large as those based on *F*, and *R*- factors based on ALL data will be even larger.

### Fractional atomic coordinates and isotropic or equivalent isotropic displacement parameters (Å^2^)

**Table:**

	*x*	*y*	*z*	*U*_iso_*/*U*_eq_	
C1	0.75411 (17)	0.59342 (9)	0.39608 (15)	0.0389 (3)	
C2	0.88807 (19)	0.63880 (10)	0.50734 (17)	0.0467 (4)	
C3	0.8400 (2)	0.68846 (12)	0.6475 (2)	0.0593 (4)	
H3	0.9278	0.7199	0.7192	0.071*	
C4	0.6643 (2)	0.69159 (11)	0.68107 (19)	0.0576 (4)	
H4	0.6344	0.7240	0.7769	0.069*	
C5	0.5313 (2)	0.64697 (10)	0.57369 (18)	0.0496 (4)	
H5	0.4122	0.6494	0.5965	0.060*	
C6	0.57723 (19)	0.59916 (9)	0.43321 (17)	0.0432 (4)	
H6	0.4874	0.5697	0.3605	0.052*	
C7	0.80781 (18)	0.54385 (10)	0.24308 (16)	0.0423 (4)	
C8	0.7418 (3)	0.36724 (13)	0.0064 (2)	0.0769 (6)	
H8A	0.8366	0.3593	0.1005	0.115*	
H8B	0.7758	0.3395	−0.0973	0.115*	
H8C	0.6338	0.3392	0.0363	0.115*	
N1	0.67678 (17)	0.50872 (10)	0.13066 (14)	0.0514 (4)	
H1A	0.565 (3)	0.5176 (12)	0.139 (2)	0.062*	
N2	0.71005 (17)	0.46382 (10)	−0.02434 (14)	0.0498 (4)	
H2A	0.805 (2)	0.4910 (11)	−0.058 (2)	0.060*	
O1	1.06320 (14)	0.63710 (9)	0.48180 (15)	0.0688 (4)	
H1B	1.071 (3)	0.6000 (15)	0.388 (3)	0.103*	
O2	0.96759 (14)	0.53627 (8)	0.21771 (13)	0.0597 (4)	

### Atomic displacement parameters (Å^2^)

**Table:**

	*U*^11^	*U*^22^	*U*^33^	*U*^12^	*U*^13^	*U*^23^
C1	0.0367 (8)	0.0413 (8)	0.0386 (7)	0.0003 (6)	0.0045 (5)	0.0015 (5)
C2	0.0368 (8)	0.0526 (9)	0.0497 (7)	0.0011 (6)	0.0014 (6)	−0.0013 (6)
C3	0.0537 (10)	0.0643 (11)	0.0576 (9)	−0.0045 (8)	−0.0034 (7)	−0.0194 (7)
C4	0.0604 (11)	0.0587 (10)	0.0547 (8)	0.0022 (8)	0.0112 (7)	−0.0166 (7)
C5	0.0436 (9)	0.0534 (9)	0.0540 (8)	−0.0020 (7)	0.0154 (6)	−0.0050 (7)
C6	0.0397 (8)	0.0466 (8)	0.0438 (7)	−0.0051 (6)	0.0064 (5)	−0.0015 (6)
C7	0.0339 (8)	0.0515 (9)	0.0422 (7)	−0.0005 (6)	0.0069 (5)	0.0013 (6)
C8	0.0874 (15)	0.0714 (14)	0.0732 (11)	0.0043 (10)	0.0149 (9)	−0.0133 (9)
N1	0.0346 (7)	0.0761 (9)	0.0442 (6)	0.0007 (6)	0.0077 (5)	−0.0167 (6)
N2	0.0402 (7)	0.0668 (9)	0.0438 (6)	−0.0031 (6)	0.0113 (5)	−0.0136 (6)
O1	0.0349 (7)	0.0969 (10)	0.0734 (7)	−0.0048 (6)	0.0013 (5)	−0.0247 (7)
O2	0.0352 (6)	0.0868 (9)	0.0586 (6)	−0.0012 (5)	0.0113 (4)	−0.0172 (5)

### Geometric parameters (Å, º)

**Table:**

C1—C6	1.3907 (19)	C6—H6	0.9300
C1—C2	1.4081 (18)	C7—O2	1.2401 (16)
C1—C7	1.4844 (18)	C7—N1	1.3336 (18)
C2—O1	1.3493 (17)	C8—N2	1.455 (2)
C2—C3	1.388 (2)	C8—H8A	0.9600
C3—C4	1.372 (2)	C8—H8B	0.9600
C3—H3	0.9300	C8—H8C	0.9600
C4—C5	1.383 (2)	N1—N2	1.4152 (16)
C4—H4	0.9300	N1—H1A	0.859 (19)
C5—C6	1.3709 (19)	N2—H2A	0.884 (18)
C5—H5	0.9300	O1—H1B	0.91 (2)
			
C6—C1—C2	118.12 (12)	C1—C6—H6	119.0
C6—C1—C7	123.34 (11)	O2—C7—N1	120.69 (12)
C2—C1—C7	118.52 (13)	O2—C7—C1	121.90 (12)
O1—C2—C3	118.09 (13)	N1—C7—C1	117.40 (12)
O1—C2—C1	122.37 (12)	N2—C8—H8A	109.5
C3—C2—C1	119.53 (14)	N2—C8—H8B	109.5
C4—C3—C2	120.61 (13)	H8A—C8—H8B	109.5
C4—C3—H3	119.7	N2—C8—H8C	109.5
C2—C3—H3	119.7	H8A—C8—H8C	109.5
C3—C4—C5	120.57 (13)	H8B—C8—H8C	109.5
C3—C4—H4	119.7	C7—N1—N2	122.75 (12)
C5—C4—H4	119.7	C7—N1—H1A	122.9 (11)
C6—C5—C4	119.14 (14)	N2—N1—H1A	113.8 (11)
C6—C5—H5	120.4	N1—N2—C8	111.07 (12)
C4—C5—H5	120.4	N1—N2—H2A	105.5 (10)
C5—C6—C1	122.00 (13)	C8—N2—H2A	111.6 (11)
C5—C6—H6	119.0	C2—O1—H1B	106.4 (14)
			
C6—C1—C2—O1	179.99 (12)	C2—C1—C6—C5	−0.2 (2)
C7—C1—C2—O1	−1.5 (2)	C7—C1—C6—C5	−178.69 (12)
C6—C1—C2—C3	−1.0 (2)	C6—C1—C7—O2	−176.57 (13)
C7—C1—C2—C3	177.58 (13)	C2—C1—C7—O2	5.0 (2)
O1—C2—C3—C4	−179.12 (15)	C6—C1—C7—N1	4.7 (2)
C1—C2—C3—C4	1.8 (2)	C2—C1—C7—N1	−173.80 (13)
C2—C3—C4—C5	−1.4 (2)	O2—C7—N1—N2	−1.9 (2)
C3—C4—C5—C6	0.2 (2)	C1—C7—N1—N2	176.89 (12)
C4—C5—C6—C1	0.6 (2)	C7—N1—N2—C8	88.87 (18)

### Hydrogen-bond geometry (Å, º)

Cg is the centroid of the C1–C6 ring.

**Table:**

*D*—H···*A*	*D*—H	H···*A*	*D*···*A*	*D*—H···*A*
O1—H1*B*···O2	0.91 (2)	1.72 (2)	2.5535 (15)	150 (2)
N1—H1*A*···N2^i^	0.859 (19)	2.16 (2)	2.9415 (18)	151.9 (15)
N1—H1*A*···N1^i^	0.859 (19)	2.619 (18)	3.1403 (18)	120.4 (13)
N2—H2*A*···O2^ii^	0.884 (18)	2.253 (17)	2.9866 (16)	140.3 (14)
C4—H4···*Cg*^iii^	0.93	2.83	3.6713 (13)	152

Symmetry codes: (i) −*x*+1, −*y*+1, −*z*; (ii) −*x*+2, −*y*+1, −*z*; (iii) *x*, −*y*+3/2, *z*+1/2.
